# Supplementary figures and images for: Inhibitory IL-10-producing CD4+ T cells are T-bet-dependent and facilitate cytomegalovirus persistence via coexpression of arginase-1
Source: eLife. 2023 Jul 13;12:e79165. doi: 10.7554/eLife.79165 (PMC10344424; doi:10.7554/eLife.79165)

Clement\_et\_al  
Figure 3C uncut gels  
Black boxes indicate lanes run

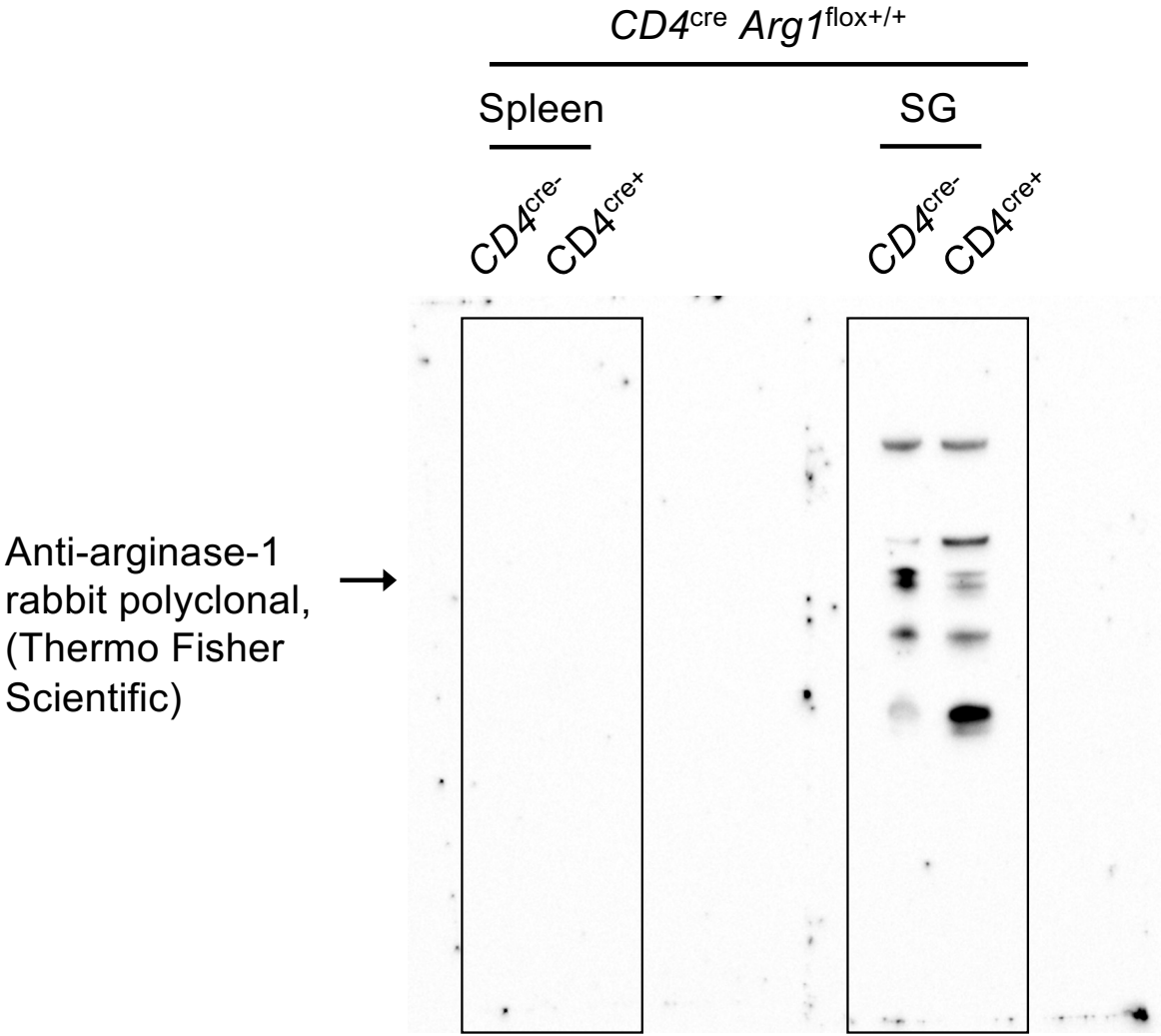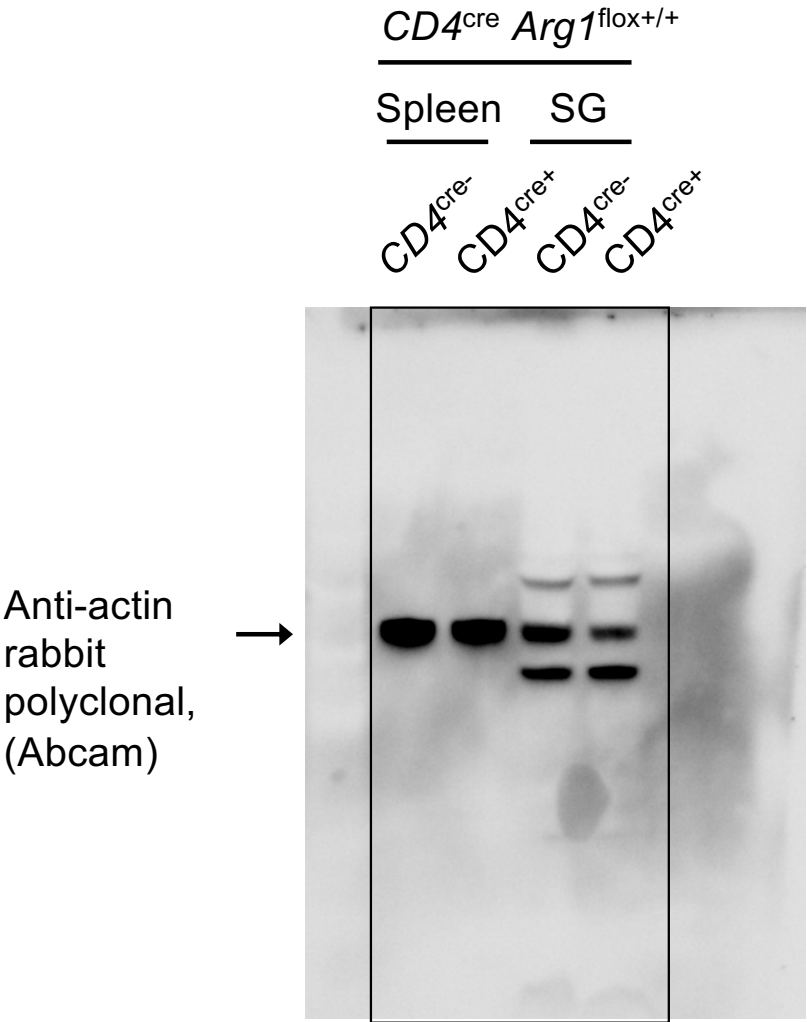

Supplement: Figure 3—source data 1. — Source data Figure 3A: expression of Arg1 among leukocytes isolated via magnetic separation from the salivary glands (SGs) and spleens of Cd4+/+Arg1flox/flox or Cd4Cre/+Arg1flox/flox mice on day 14 p.i. detected by Western blot. Original blots are shown for each antibody. Uncut blots are shown with black boxes to delineate the images used in Figure 3A. [file elife-79165-fig3-data1.zip › Figure3_Source_Data/Figure3_Source_Data_1/Clement_et_al_Fig3A_source_data_uncut_gels.pdf]

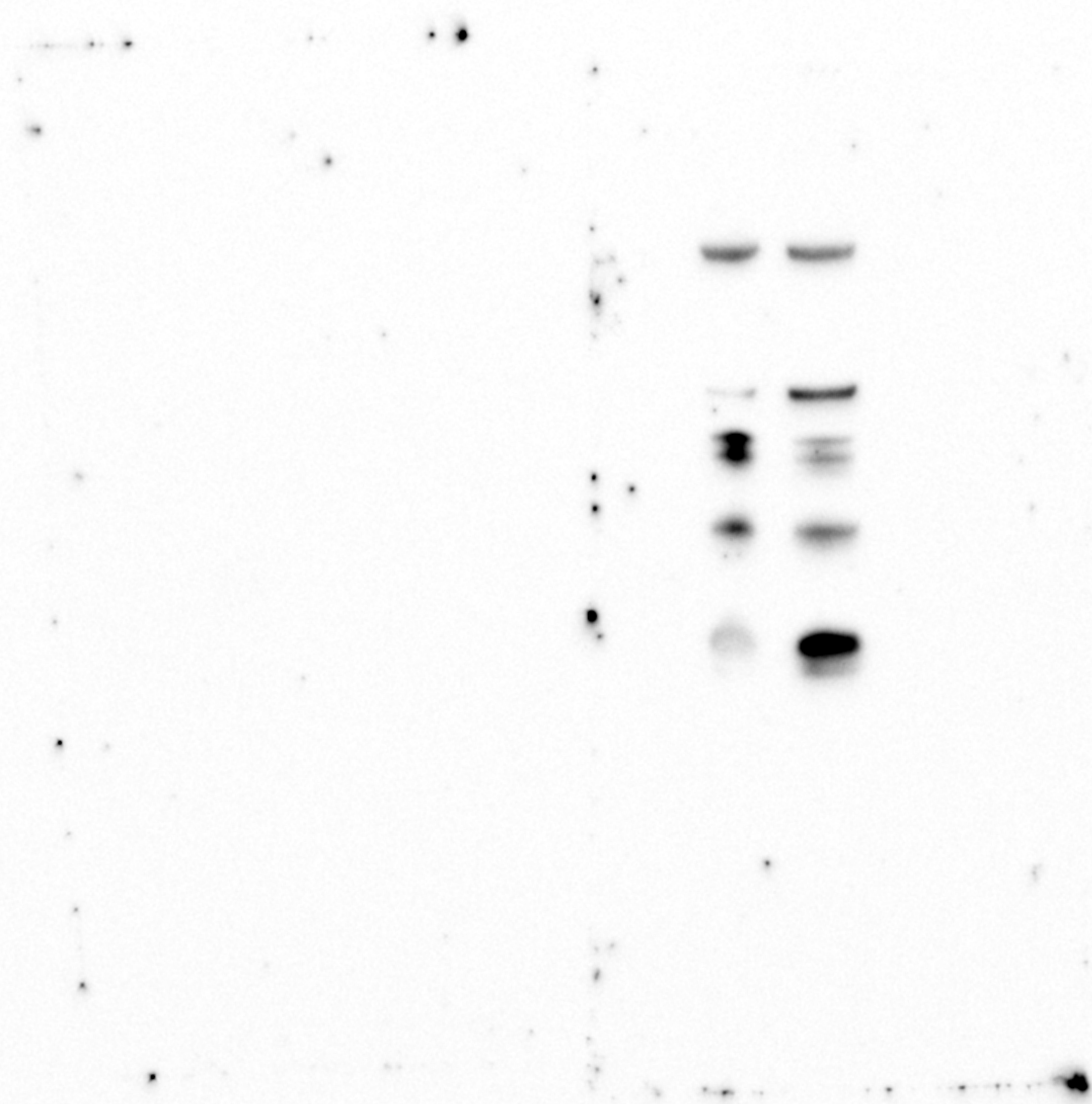

Supplement: Figure 3—source data 1. — Source data Figure 3A: expression of Arg1 among leukocytes isolated via magnetic separation from the salivary glands (SGs) and spleens of Cd4+/+Arg1flox/flox or Cd4Cre/+Arg1flox/flox mice on day 14 p.i. detected by Western blot. Original blots are shown for each antibody. Uncut blots are shown with black boxes to delineate the images used in Figure 3A. [file elife-79165-fig3-data1.zip › Figure3_Source_Data/Figure3_Source_Data_1/Clement_et_al_Fig3A_source_data_original_blot_Arg1.pdf]

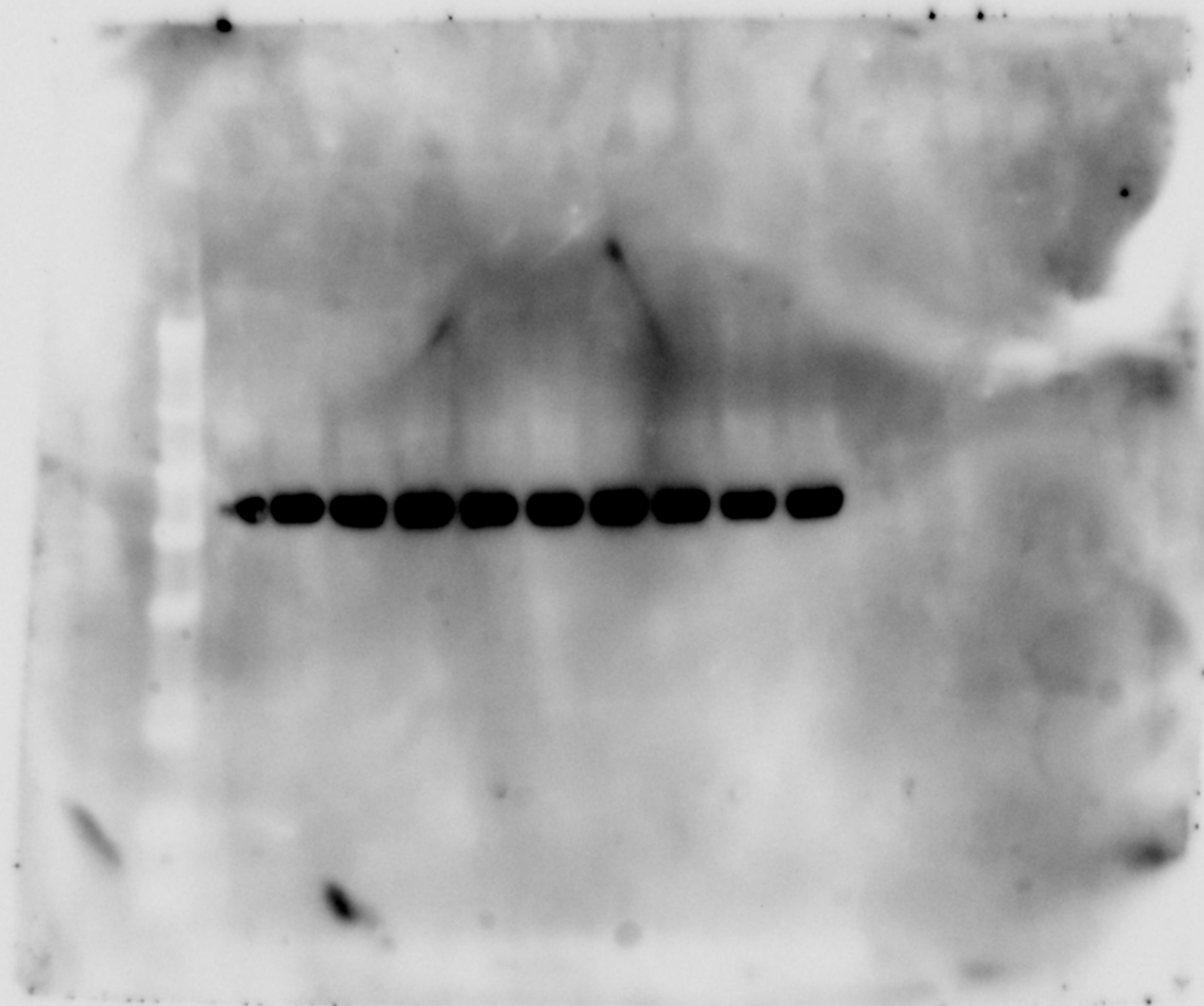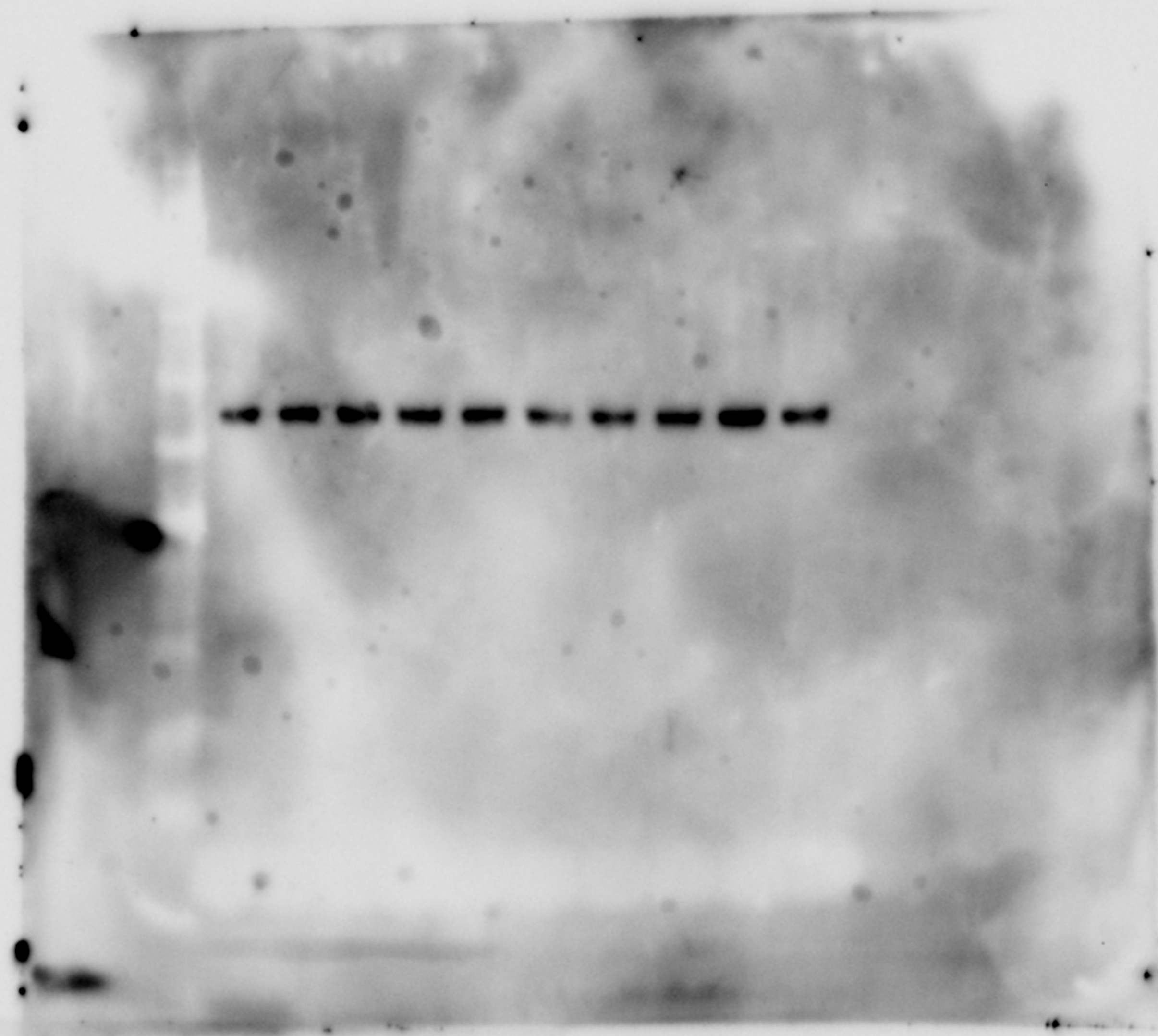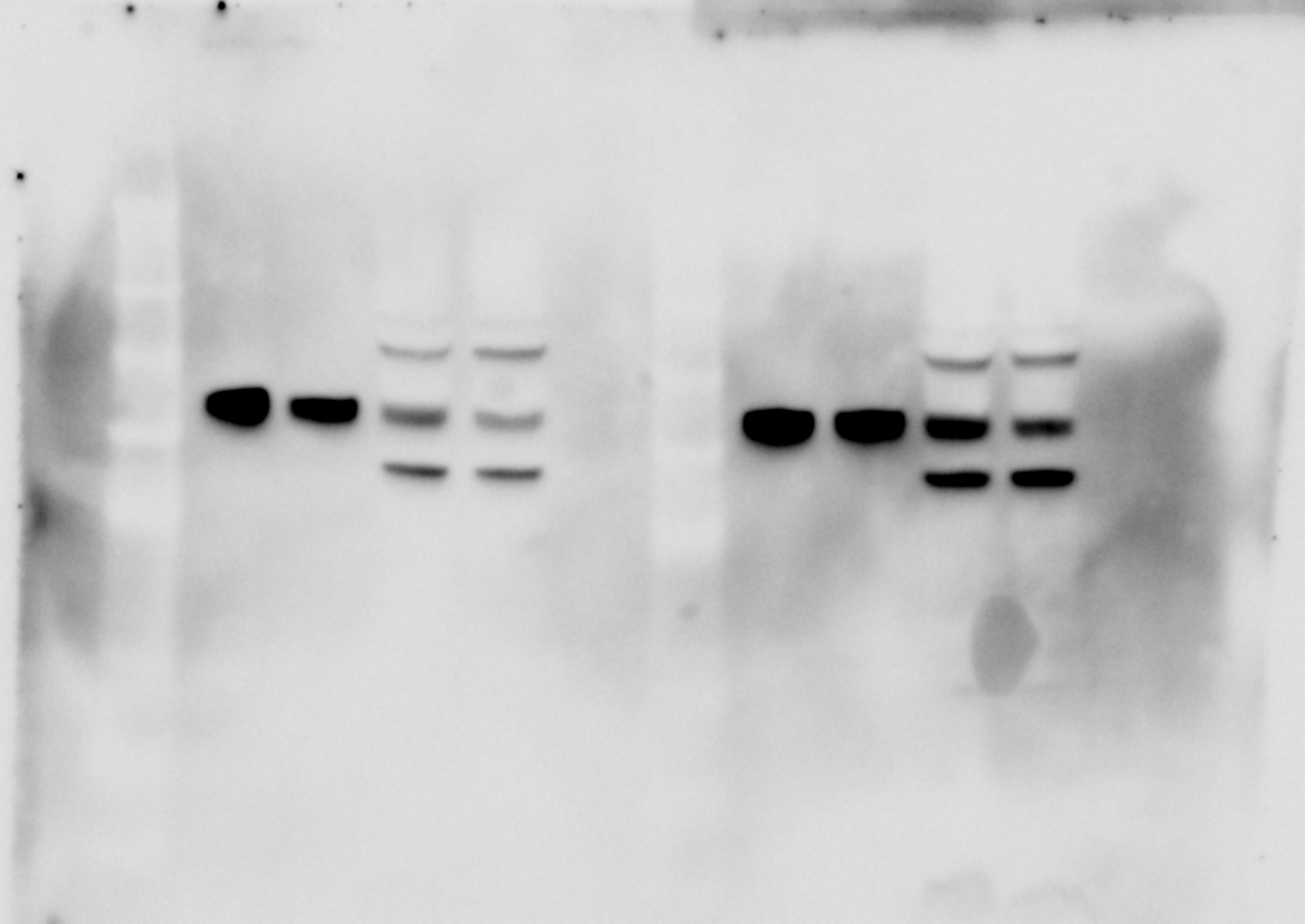

Supplement: Figure 3—source data 1. — Source data Figure 3A: expression of Arg1 among leukocytes isolated via magnetic separation from the salivary glands (SGs) and spleens of Cd4+/+Arg1flox/flox or Cd4Cre/+Arg1flox/flox mice on day 14 p.i. detected by Western blot. Original blots are shown for each antibody. Uncut blots are shown with black boxes to delineate the images used in Figure 3A. [file elife-79165-fig3-data1.zip › Figure3_Source_Data/Figure3_Source_Data_1/Clement_et_al_Fig3A_source_data_original_blot_actin.pdf]
